# Special Issue “Novel Antibacterial Agents”

**DOI:** 10.3390/ph14040382

**Published:** 2021-04-19

**Authors:** Fiorella Meneghetti, Daniela Barlocco

**Affiliations:** Department of Pharmaceutical Sciences, University of Milan, Via L. Mangiagalli 25, 20133 Milan, Italy; daniela.barlocco@unimi.it

This Special Issue of *Pharmaceuticals* is devoted to significant advances achieved in the field of antibacterial agents. Here, we report recent efforts made to develop new antimicrobials with novel modes of action/resistance, and offer perspectives on the future directions of antibacterial agents.

Antimicrobial resistance has become a major threat to global health and the twenty-two published articles, included here, evidence that the discovery and development of new antibiotics is extremely challenging.

This Special Issue is focused on the search for new chemical entities, starting from both natural and synthetic compounds and addressing different targets. In addition, recent findings are presented and discussed, highlighting strategies of fighting bacterial resistance.

Investigation into antimicrobials covers a wide research area, as emphasized in this Special Issue, spanning from the design, synthesis, and characterization of new compounds, supported by molecular modeling techniques, to the development of biological tests. This is all possible thanks to the contributions of experts.

Natural products, as rich sources of chemical diversity, offer excellent possibilities to identify novel leads in medicinal chemistry. In this direction, S. Garzoli et al. [1], via Headspace-Gas Chromatography/Mass Spectrometry (HS-GC/MS), identified 28 components, mainly belonging to the monoterpenes family, in the essential oils from needles (EOs) of four *Pinaceae*. Both the liquid and vapour phases were evaluated for their antibacterial activity against three Gram-negative (*Escherichia coli*, *Pseudomonas fluorescens*, and *Acinetobacter bohemicus*) and two Gram-positive (*Kocuria marina* and *Bacillus cereus*) bacteria using different assays. Better results were obtained with the vapour phase. In addition, a concentration-dependent antioxidant activity was evidenced for all the EOs. The Authors highlighted the importance of α-pinene as a novel natural antibacterial agent.

Other interesting antioxidant compounds were identified by L.C. Nascimento da Silva et al. [2], in *B. tetraphylla* leaf methanolic extracts (BTME) which, when tested on *Tenebrio molitor* larvae inoculated with heat-inactivated *E. coli*, proved to be able to protect the larvae from the stress. A mixture of aliphatic (terpenes, fatty acids, carbohydrates) and aromatic compounds (phenolic derivatives) were evidenced in BTME using NMR analysis.

Antioxidant properties, in addition to antimicrobial and cytotoxic activity, were reported by A.J.L. Pombeiro et al. [3] in a series of saccharin-tetrazolyl and -thiadiazolyl derivatives. The best antioxidant results were shown for (*N*-(1-methyl-2*H*-tetrazol-5-yl)-*N*-(1,1-dioxo-1,2-benzisothiazol-3-yl) amine, while all the compounds had insignificant toxicity when tested in an *Artemia salina* model.

Several EOs, derived from *Origanum majorana*, *Rosmarinus officinalis*, and *Thymus zygis* medicinal plants were investigated by A. Gaber et al. [4] for their ability to inhibit biofilm formation and eradicate methicillin-resistant *Staphylococcus aureus* (MRSA) isolates. The best activity was found in *T. Zygis*, but all the studied EOs showed interesting properties (the percentage of inhibition ranging from 10.20 to 95.91%, and percentage of eradication ranging from 12.65 to 98.01%), thus, representing potential alternatives to antibiotics.

The inhibition of biofilm formation was also studied also by B. Citterio et al. [5], who investigated the properties of the marine bisindole alkaloid 2,2-bis(6-bromo-1*H*-indol-3-yl)ethanamine and its fluorinated analogue which were tested both for their potential use as antibiotic adjuvants and antibiofilm agents against *S. aureus* CH 10850 (MRSA) and *S. aureus* ATCC 29213 (MSSA). The fluorinated derivative showed a higher potency in eradicating a preformed biofilm. Both compounds showed a safe profile and were indicated for in vivo application as adjuvants to restore antibiotic treatment against MRSA.

Biofilm formation is an urgent problem in dentistry, due to the high porosity and absorptiveness of the polymers commonly used for the 3D-printed dental prostheses. D.-H. Lee et al. [6] studied the effects of a new chlorhexidine (CHX)-loaded polydimethylsiloxane (PDMS)-based coating material on the surface microstructure, surface wettability and antibacterial activity of 3D-printing dental polymer. The antibacterial was first encapsulated in mesoporous silica nanoparticles (MSN). The significant experimental results supported this approach.

Functionalization of oxidized multi-walled carbon nanotubes (oxCNTs) was studied by Z. Sideratou et al. [7], who used a simple non-covalent modification procedure, using hyperbranched poly(ethyleneimine) derivatives (QPEIs), with various quaternization degrees. The aqueous dispersion of this material was found to be stable over 12 months and to be provided with significant antibacterial and anti-cyanobacterial activity. It was suggested by the Authors for application in the disinfection industry.

W.Y. Bang et al. [8] proposed the use of the cell-free supernatant from *Lactobacillus plantarum* NIBR97 as an alternative to chemical disinfectants. By scanning electron microscopy (SEM), this was shown to cause cellular lysis in bacterial membranes, thus, indicating the involvement of peptides or proteins in its mechanism of action and suggesting the use of proteinase K treatment to support its antibacterial activity. In addition, it showed good antiviral properties, possibly through a different mechanism of action.

A study by A. Baycal et al. [9] on *Moringa oleifera* reported the correlation between the capability of laser-induced breakdown spectroscopy (LIBS) to monitor the elemental compositions of plants and their biological effects. In particular, the bioactive components of the seed (MOS) alcoholic extract were tested against *Escherichia coli* and *Staphylococcus aureus* via an agar well diffusion (AWD) assay and scanning electron microscopy (SEM). An interesting activity against Gram-positive bacteria was evidenced. In addition, the authors reported that MOS extract exhibited significant inhibitory properties on HCT116 cell growth, whereas no effects were noticed in a parallel assay on HEK-293 cells. According to the authors, the antibacterial and anticancer potency of the MOS extract could be attributed to several complexes, e.g., ethyl ester and D-allose and hexadecenoic, oleic and palmitic acids.

J.-H Moon et al. [10] reported their results on 3-pentylcatechol (PC), a synthetic urushiol derivative, able to inhibit the growth of *Helicobacter pylori* in an in vitro assay, in comparison with triple therapy (omeprazole, metronidazole, and clarithromycin). The authors report that PC displayed better effects than triple therapy at all doses. Interestingly, synergism was shown when PC and triple therapy were co-administered.

The composition and biological properties of *Thymus mastichina,* a diffuse semishrub of jungles and the rocky landscapes of the Iberian Peninsula, were reviewed by A.R.T.S. Araujo et al. [11]. Several properties were reported both for the extracts and the essential oils. In particular, activity against methicillin resistant bacteria and antifungal activity should be highlighted, though several other properties (e.g., anticancer, antiviral, insecticidal, repellent, anti-Alzheimer’s, and anti-inflammatory) have been investigated. The authors suggest that *Thymus mastichina* should be considered for use in food and cosmetic applications other than in the pharmaceutical field.

Antibiotic resistance was also the target of the paper by M. Sessevmez et al. [12], who indicated bacteriophages as an alternative method, which was first proposed in the early 20th century by d’Herelle, Bruynoghe and Maisin to treat bacterial infections. Different administration methods are possible, including topical application, inhalation, oral or parenteral delivery. *Pseudomonas aeruginosa*, *Mycobacterium tuberculosis* and *Acinetobacter baumannii*, responsible for the main drug resistant infections, are potential targets of phages, which are developed under a strict quality control regime.

Tuberculosis was also the target of F. Meneghetti et al. [13], who described novel furan derivatives as inhibitors of salicylate synthase MbtI, an essential enzyme of mycobacterium, absent in human cells. The best compound, which provided comparable inhibitory properties to the previous leads but endowed a better antitubercular activity, was 5-(3-cyano-5-(trifluoromethyl)phenyl)furan-2-carboxylic acid, which will form the basis of future studies.

Covalent inhibitors of another bacterial enzyme for which there is no human orthologue, namely UDP-*N*-acetylglucosamine enolpyruvyl transferase (MurA), have been developed by G.M. Keserü et al. [14], who indicated bromo-cyclobutenaminones as novel electrophilic probes by screening a small library of cyclobutenone derivatives. The bromine atom has been recognized as an essential requirement, and MS/MS experiments have led to the suggestion that Cys115 is also involved. The stability and bioavailability of these compounds was also assessed.

Another alternative to the use of antibiotics in bacterial infections was proposed by F.M. Goycoolea et al. [15] who studied a library of 23 pure compounds of different chemical structures, assessing their quorum sensing (QS) inhibition activity. The best results were obtained with phenazine carboxylic acid (PCA), 2-heptyl-3-hydroxy-4-quinolone (PQS), 1*H*-2-methyl-4-quinolone (MOQ) and genipin, which exhibited QS inhibition activity without compromising bacterial growth.

A specific disease, characterized by complex aetiological mechanisms, namely atopic dermatitis (AD), was the topic of a review by R. Di Marco et al. [16]. Loss of the skin barrier, linked to dysbiosis and immunological dysfunction, which causes an imbalance in the ratio between the pathogen *Staphylococcus aureus* and/or other microorganisms residing in the skin, is considered as a crucial factor contributing to AD. Though the review suggests several treatments for the disease, including the use of bacteria and/or microbiota transplantation, together with different drug delivery systems, the authors conclude that a standardized process is necessary in order to obtain reliable data.

F. Sparatore et al. [17] addressed the topic of Leishmaniases, the therapy of which is presently based on expensive drugs which are associated with severe side-effects and the treat of resistance. They tested sixteen lucanthone and four amitriptyline analogues in vitro, all of which were characterized by a bulky quinolizidinylalkyl moiety, against *Leishmania tropica* and *L. infantum* promastigotes. All compounds displayed significant activity (IC_50_ in the low µM range) and low cytotoxicity. The authors suggest that these analogues could act through trypanothione reductase (TryR) inhibition.

In the search for selective agents that are capable of treating bacterial diarrhoea without affecting the host intestinal microbiota, L. Kokoska et al. [18] investigated ten phytochemicals and their synthetic analogues (berberine, bismuth subsalicylate, ferron, 8-hydroxyquinoline, chloroxine, nitroxoline, salicylic acid, sanguinarine, tannic acid, and zinc pyrithione) in vitro and compared the results with six commercial antibiotics (ceftriaxone, ciprofloxacin, chloramphenicol, metronidazole, tetracycline, and vancomycin) against 21 intestinal pathogenic/probiotic (e.g., *Salmonella* spp. and *bifidobacteria*) bacterial strains and three intestinal cancer/normal (Caco-2, HT29, and FHs 74 Int) cell lines. Several compounds, e.g., chloroxine, ciprofloxacin, nitroxoline, tetracycline, and zinc pyrithione exhibited potent selective growth-inhibitory activity against pathogens, while 8-hydroxyquinoline and sanguinarine provided the best activity towards cancer cells. It should also be noted that none of the compounds were found to be cytotoxic to normal cells. Once more, plants have been suggested as a promising source for novel drugs.

N. D’Amelio et al. [19] investigated the mechanism of action of a synthetic peptide (K11), whose antibacterial properties have been previously reported. Via a liquid and solid-state NMR technique, they studied the interaction of K11 with different biomimetic membranes and reported that this can destabilize them. In addition, via molecular dynamic simulations, they suggested that K11 could penetrate the membranes in four steps (anchoring, twisting, helix flip, and internalization) involving several lysine residues.

Additionally, also within the field of synthetic compounds, B. Altava et al. [20] investigated how they could vary the antibacterial properties of imidazole and its imidazolium salts derived from *L*-valine and *L*-phenylalanine, by modulating their lipophilicity. When tested on *E. Coli* and *B. Subtilis* bacterial strains, very encouraging results were obtained. In particular, the minimum bactericidal concentration (MBC) of one compound towards *B. subtilis* was found to be lower than the IC_50_ cytotoxicity value for the control cell line, HEK-293. Moreover, the capability of these structures to aggregate in different media was investigated to establish the importance of the monomeric species.

The effects of substituent variation on a different substrate, namely *tris*(1*H*-indol-3-yl)methylium, was investigated by A.S. Trenin et al. [21]. The synthesized compounds were tested on 12 bacterial strains that were either sensitive or resistant to meticillin. The results indicated that antibacterial properties depended on the chain length, with the best activity residing in compounds with C5–C6 chains. The most interesting compounds showed better antibacterial properties than levofloxacin on MRSA and had a very safe profile.

Antibacterial and antifungal activity was investigated in vitro by P. Eleftheriou et al. [22] on a series of 3-amino-5-(indol-3-yl) methylene-4-oxo-2-thioxothiazolidine derivatives. Compounds exhibited significant activity both on Gram-positive and Gram-negative bacteria, demonstrating a potency greater than ampicillin. Similarly, their antifungal activity was superior to that of ketoconazole. Docking studies have suggested that their antibacterial activity could be derived from *E. coli* Mur B inhibition, while CYP51 inhibition would be responsible for the antifungal activity.

The research described in the articles constituting this Special Issue collectively provides extremely useful examples of the results that have been recently achieved in the field of antibacterial drug development.

We hope the readers enjoy this Special Issue and are inspired to develop new approaches for antibacterial disease diagnosis, treatment and to circumvent resistance issues.
